# Enrichment of the TRIM21-IgG complex in placental villi during HIV-1 infection

**DOI:** 10.3389/fimmu.2026.1922263

**Published:** 2026-09-18

**Authors:** Beatriz Noronha Campo, Emanuella Sarmento Alho de Sousa, Nátalli Zanete Pereira, Anna Julia Pietrobon, Helaine Maria Besteti Pires Mayer Milanez, Mirian Nacagami Sotto, Gabriela Domingues Mattos, Gabriel Benevides Simões, Daniel Pereira Sousa, Marina de Mello Naresse, Vinícius Nunes Cordeiro Leal, Maria Notomi Sato

**Affiliations:** 1 Laboratory of Medical Investigation 56, Department of Dermatology, University of São Paulo Medical School, Institute of Tropical Medicine, São Paulo, Brazil; 2 Department of Tocoginecologia, State University of Campinas, Campinas, Brazil; 3 Laboratory of Medical Investigation 50, Department of Pathology, University of São Paulo Medical School, São Paulo, Brazil; 4 Department of Immunology, Institute of Biomedical Sciences, University of São Paulo, São Paulo, Brazil

**Keywords:** antiretroviral therapy, HIV-1, host restriction factors, innate immunity, maternal-fetal interface, placenta, TRIM21

## Abstract

**Introduction:**

Innate immunity constitutes a pivotal defense mechanism protecting the maternal-fetal interface against viral pathogens. However, the dynamics of placental immunity in pregnant women with chronic HIV-1 infection undergoing suppressive antiretroviral therapy (ART) remain poorly understood. Tripartite motif (TRIM) family proteins act as critical host restriction factors or indirect regulators of antiviral pathways through ubiquitination cascades.

**Objectives:**

In this study, we profiled the transcriptional expression of prominent antiviral *TRIM *genes − namely *TRIM5α, TRIM8, TRIM21, TRIM22, TRIM39, TRIM64B, * and *TRIM71* − in both the decidual and chorionic villi compartments of HIV-1-infected parturient women with undetectable viral loads compared to healthy controls (HC).

**Methods:**

Quantitative real-time PCR in the decidua and villi was performed in both the decidual and chorionic villi compartments of HIV-1-infected parturient women compared to HC. Gene network was generated using GeneMANIA was assessed to identify functionally related genes. Given that TRIM21 classically intercepts antibody-coated target complexes, we evaluated its spatial intersection with maternal humoral components via immunofluorescence using a TissueGnostics scanning confocal microscope.

**Results:**

Quantitative real-time PCR revealed compartment-specific alterations, with a significant upregulation of *TRIM21, TRIM39*, and *TRIM64B* in the decidua, and *TRIM21*, and *TRIM39* in the chorionic villi of the HIV-1 group. Systems biology and functional network analyses integrated these central hubs (*TRIM21, TRIM39* and *TRIM64B*) into pathways governing the innate immune response, multi-step protein ubiquitination, type I interferon signaling, response to virus and the regulation of cytokine production. Notably, TRIM21 and IgG protein expressions, along with their physical intracellular colocalization, were significantly enriched exclusively within the placental villi of HIV-1-infected mothers.

**Conclusions:**

Taken together, our findings unveil a localized upregulation of antiviral *TRIM *factors at the maternal-fetal interface and highlight a novel enrichment of TRIM21–IgG complexes within the chorionic villi. This specialized intracellular synergy between innate and humoral mechanisms opens promising avenues for therapeutic and protective strategies against vertical viral transmission.

## Introduction

The global HIV epidemic affects approximately 40.9 million people living with HIV (PWH), 53% of whom are women and girls (1). This demographic distribution underscores the critical concern regarding vertical (mother-to-child) viral transmission. Although antiretroviral therapy (ART) is remarkably effective in preventing perinatal HIV-1 transmission, the rate of HIV-1 transmission from a mother living with the virus to her child during pregnancy, childbirth, or breastfeeding is approximately 2% (2). However, in the absence of intervention, transmission can range from 15% to 45% (3). This relatively low baseline transmission rate highlights the importance of investigating innate immunity at the maternal-fetal interface.

The placenta plays a pivotal role in facilitating maternal-fetal interactions by balancing immunity and tolerance, thereby supporting fetal development and providing protection against harmful pathogens. Our group has previously demonstrated that the placenta maintains an effective local antiviral state associated with a suppressed inflammatory environment, which preserves maternal immune homeostasis (4, 5). Specifically, restriction factors such as apolipoprotein B mRNA-editing enzyme 3G (A3G) and A3F, tripartite motif family-5α (TRIM5α), TRIM22, myxovirus resistance protein A (MxA), stimulator of interferon genes (STING) (5), and type I/III interferons (IFNs) − along with their signaling molecules, IRF3 and IRF7 − (4) were found to be upregulated in the placentas of HIV-infected mothers compared to uninfected mother-newborn pairs. This upregulation occurred preferentially within the villous compartment. Conversely, while inflammatory factors such as cytokines and inflammasome components were present, they appeared to be dampened in placental tissues from PWH (4).

Tripartite motif (TRIM) proteins belong to the E3 ubiquitin ligase family and regulate a wide range of signaling pathways involved in immune responses (6). TRIMs participate in various cellular processes, including proliferation, differentiation, development, oncogenesis, and apoptosis. Ubiquitination represents one of the major post-translational modification mechanisms in eukaryotes. To date, more than eighty TRIM proteins have been identified in humans, spanning almost all multicellular animals. Structurally, TRIMs are characterized by an N-terminal region containing three conserved domains: a RING finger, one or two B-box domains, and a coiled-coil region (7). These proteins can mediate protein ubiquitination and chromosome translocation by modulating signaling pathways such as p53, NF-κB, AKT, MAPK, and Wnt/β-catenin, among other molecular regulatory mechanisms (8). Crucially, the immunological functions of TRIM proteins are exerted either by directly interfering with key steps of the viral life cycle or by acting as indirect regulators of antiviral cellular signaling (9, 10).

For instance, TRIM5α restricts retroviral capsids by interacting with determinants in assembled capsid antigen structures via its C-terminal SPRY domain (11), playing a key role in the type I IFN-mediated suppression of HIV-1 replication (12). TRIM22 controls viral replication and plays a more complex role during chronic infection by establishing or maintaining HIV-1 latent reservoirs (13). TRIM21 acts as an intracellular antibody receptor that intercepts antibody-coated viruses that have evaded extracellular neutralization, thereby coordinating viral neutralization (14). However, TRIM21 also induces the proteasomal degradation of IRF7 (15) and IRF3 (16), the autophagic degradation of IRF3 (17) and the degradation of the dNTPase SAMHD1 during enterovirus 71 infection (18). Furthermore, TRIM71 interacts actively with viral or retroviral machinery through specific cellular pathways, such as CSNK2/Autophagy (19). Other TRIMs regulate the NF-κB pathway, such as TRIM39 (20), or enhance host JAK-STAT signaling, such as TRIM8, which binds to Suppressor of Cytokine Signaling 1 (SOCS1) (21) and protects regulatory proteins (e.g., IRF7) from degradation to induce IFN production upon HIV infection (22). Lastly, TRIM64B regulates autophagy receptors, activating a feedback loop with NF-κB via IκBα ubiquitination (23).

Despite these insights, a key aspect of TRIM biology remains relatively unexplored within the placental microenvironment of women with controlled HIV-1 viral load. In this study, we evaluated the expression of specific antiviral TRIMs − namely TRIM5α, TRIM21, TRIM22, TRIM8, TRIM64B, TRIM39, and TRIM71 − in both the villous and decidual placental compartments of HIV-1-infected parturient women compared to healthy controls. Among these, TRIM21 stands out as a particularly intriguing factor to explore as a potential player in placental protection during HIV-1 infection.

## Results

### Placentas from HIV-1-infected mothers exhibit increased maternal vascular malperfusion features with well-preserved inflammatory status

**Table 1** shows the clinical and demographic characteristics of the HIV-1-infected women who gave birth. All patients were receiving ART treatment. Five out of 15 had a detectable viral load, four of whom had a high viral load. One of these (H14, 31 copies/mL) was considered to have a high viral load because their previous viral load was >6,000 copies/mL. An undetectable viral load (<50 copies/mL) was observed in 10 out of 15 patients. One of the newborns became infected via vertical transmission (H7).

**Table 1:** Clinical and demographic characteristics of the HIV-1 infected participants.

ID	Day of delivery	ART	VL	diagnostic time	Adhesion time	therapy at the time of delivery	Outcome
H1	28/08/19	ABC, 3TC, ATV and RTV	Und	Born via VT	NR	NR	NR
H2	20/02/18	TDF, 3TC and EFV	Und	2012	NR	NR	NR
H3	02/05/19	TDF, 3TC and NVP	Und	NR	NR	NR	NR
H4	02/05/23	AZT, TDF, 3TC and DTG	Und	NR	NR	NR	NR
H5	30/10/17	TDF, 3TC and EFV	1.582**	2010	NR	NR	NR
H6	27/01/20	TDF, 3TC and EFV	Und	NR	NR	NR	NR
H7	14/03/19	RAL, TDF and 3TC	20609**	2019	During last 1 month of pregnancy	Yes	VT
H8	25/08/23	TDF, 3TC, DRV and RTV	Und	2012	NR	NR	NR
H9	07/11/23	TDF, 3TC and DTG	25*	2023	During last 6 months of pregnancy	Yes	No VT
H10	09/04/24	TDF, 3TC and DTG	Und	NR	all pregnancy	No	No VT
H11	12/08/24	TDF, 3TC and DTG	Und	2013	all pregnancy	No	Lost follow-up (monitored up to the 6th month)
H12	04/03/24	TDF, 3TC, DTG, DRV and RTV	Und	NR	all pregnancy	No	Lost follow-up (no monitored)
H13	27/03/24	TDF, 3TC and DTG	Und	NR	all pregnancy	No	No VT
H14	27/06/24	TDF, DRV, 3TC and DTG	31**	Born via VT	During last 7 months of pregnancy	Yes	Lost follow-up (monitored up to the 6th month)
H15	17/02/20	TDF, 3TC and RAL	383**	2019	NR	NR	NR

ID, identification number; H, HIV-1; NR, not reported; ART, antiretroviral therapy; ABC, abacavir; 3TC, lamivudine; ATV, atazanavir; EFV, efavirenz, NVP, nevirapine, DRV, darunavir, RTV, ritonavir; DTG, dolutegravir; TDF, tenofovir; RAL, raltegravir; Und: undetected (<50 copies/mL), VL, viral load (copies/mL); VT: vertical transmission; *, Low viral load; **, High viral load.

Histopathological examination of placental tissues from both HIV-1-infected (n=6) and uninfected parturient women (n=6) revealed no inflammatory alterations, such as acute or chronic chorioamnionitis or villitis (**Table 2**). Maternal vascular malperfusion (MVM) is typically characterized by features including low placental weight, thin umbilical cords, accelerated villous maturation, distal villous hypoplasia, increased syncytial knots, decidual arteriopathy, and retroplacental hemorrhage (24). In this cohort, MVM features were primarily driven by villous hypoplasia and syncytial knots. Syncytial knots were detected in 66.7% of the HIV-1 group versus 33.3% of the control group, while villous hypoplasia was observed in 83.3% versus 50.0%, respectively, demonstrating a higher frequency in HIV-infected mothers (**Figure 1**). No comorbidities were reported in the health control group (**Table 2**), where the indication for elective cesarean delivery was a history of a previous C-section.

**Table 2:** Demographic characteristics of the parturient and newborn.

ID	Age	WG	Comorbidities	PW	NBW	NBS
HC 1*	45	40.2	Absent	NR	3640	F
HC 2	37	N/D	Absent	NR	3340	F
HC 3*	38	38.2	Anemia and UTI	580	3870	M
HC 4*	32	38.3	Absent	NR	3230	F
HC 5*	34	38.1	Absent	720	2630	F
HC 6	28	39	Absent	NR	3030	F
HC 7*	37	39.2	Absent	NR	3765	M
HC 8*	28	41	GDM and PE	820	4285	M
HC 9	37	39.1	Absent	NR	4300	M
HC 10	34	39.2	Absent	720	2805	M
HC 11	36	39.2	Absent	380	3650	F
HC 12	27	39.3	Absent	670	3200	F
HC 13	36	39.1	Absent	455	3330	M
HC 14	36	38.6	Absent	565	3390	M
HC 15	28	38.6	Absent	NR	3360	F
HC 16	22	40.2	Absent	585	3445	M
HC 17	31	39	Absent	590	3500	F
HC 18	33	39.1	Absent	640	3395	M
H1*	19	39.1	Absent	400	2580	F
H2*	35	NR	Absent	NR	2965	F
H3	39	39.0	Absent	665	3695	M
H4*	40	39.0	Absent	NR	3740	M
H5*	27	NR	CMV and Rubella IgG	NR	NR	NR
H6*	39	35.1	Absent	300	2080	F
H7*	22	39.0	Absent	NR	3050	M
H8	28	36.5	AD, GDM and Hypothyroidism	560	3145	M
H9	31	40.6	Absent	630	3115	F
H10	22	39.1	GDM	485	3095	F
H11	34	37.2	Absent	520	3513	M
H12	27	38.6	Absent	785	3850	M
H13	38	38.0	Absent	560	3165	M
H14	24	39.1	Absent	530	2525	F
H15	27	38.6	Toxoplasmosis IgG	465	3130	M

ID, identification number; HC, healthy group; H, HIV-1; WG, weeks of gestation (weeks); PW, placental weight (g); NBW, newborn weight (g); NBS, newborn sex; F, female; M, male; HC, UTI, urinary tract infection; CMV, Cytomegalovirus; GDM, Gestational diabetes mellitus; AD, Atopic dermatitis; PE, pre-eclampsia; NR, not reported. Data not reported (NR) refers to information missing from the medical records of pregnant women.* This indicates cases from histopathological analysis.

**Figure 1:**
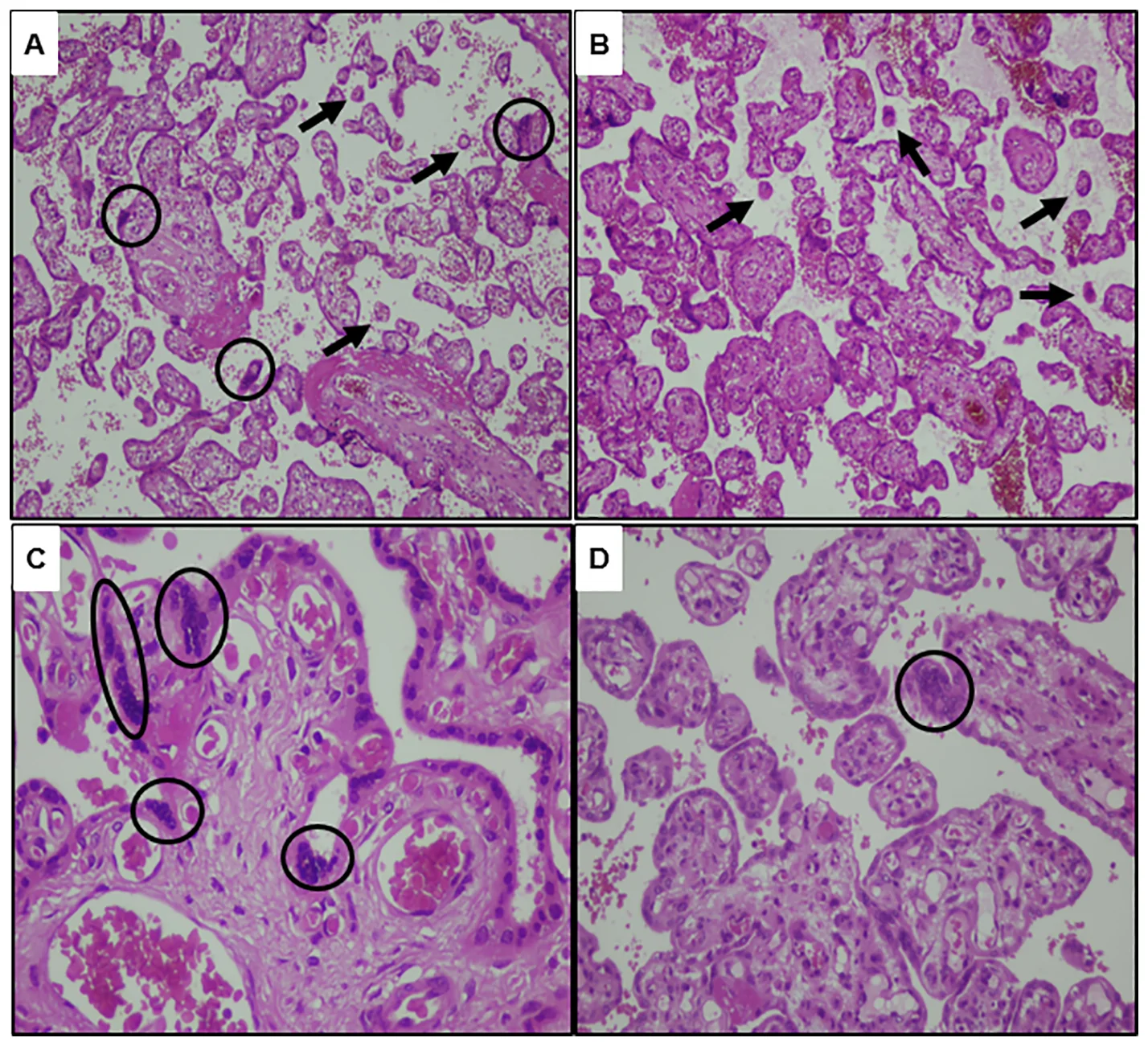
Representative placental histopathology from HIV-1-infected and uninfected mothers. Placental sections from the HIV-1-infected group (n=6) and healthy control group (n=6) were analysed by Hematoxylin and eosin (H&E) stain. **(A, C)** Placental sections from the HIV-1-infected group demonstrating features of maternal vascular malperfusion (MVM), including distal villous hypoplasia (black arrows) and syncytial knots (black circles). **(B, D)** Placental sections from healthy controls showing normal parenchymal architecture, with fewer syncytial knots and typical villous development. Pronounced intervillous spacing associated with hypoplasia is highlighted in the affected tissues. Original magnifications: **(A, B)** 10× objective and **(C, D)** 40× objective.

### Placental tissue reveals compartment-specific upregulation of antiviral TRIM restriction factors in the HIV-1 group

Emerging evidence indicates that while certain TRIM family proteins act as potent antagonists of viral replication, others may facilitate viral replication by ubiquitinating and degrading specific innate immune signaling intermediates. Given the roles of TRIMs in placental biology and viral infections, we evaluated the expression of *TRIM5α*, *TRIM8*, *TRIM21*, *TRIM22*, *TRIM39*, *TRIM64B*, and *TRIM71* via RT-qPCR in samples from HIV-1-infected mothers (n = 15) and healthy controls (HC, n = 18). In the decidua, *TRIM21*, *TRIM39*, and *TRIM64B* were significantly upregulated in the HIV-1 group compared to HCs (**Figure 2A**). Similarly, significant upregulation of *TRIM21*, and *TRIM39* was observed in the chorionic villi of HIV-1-infected mothers (**Figure 2B**).

**Figure 2:**
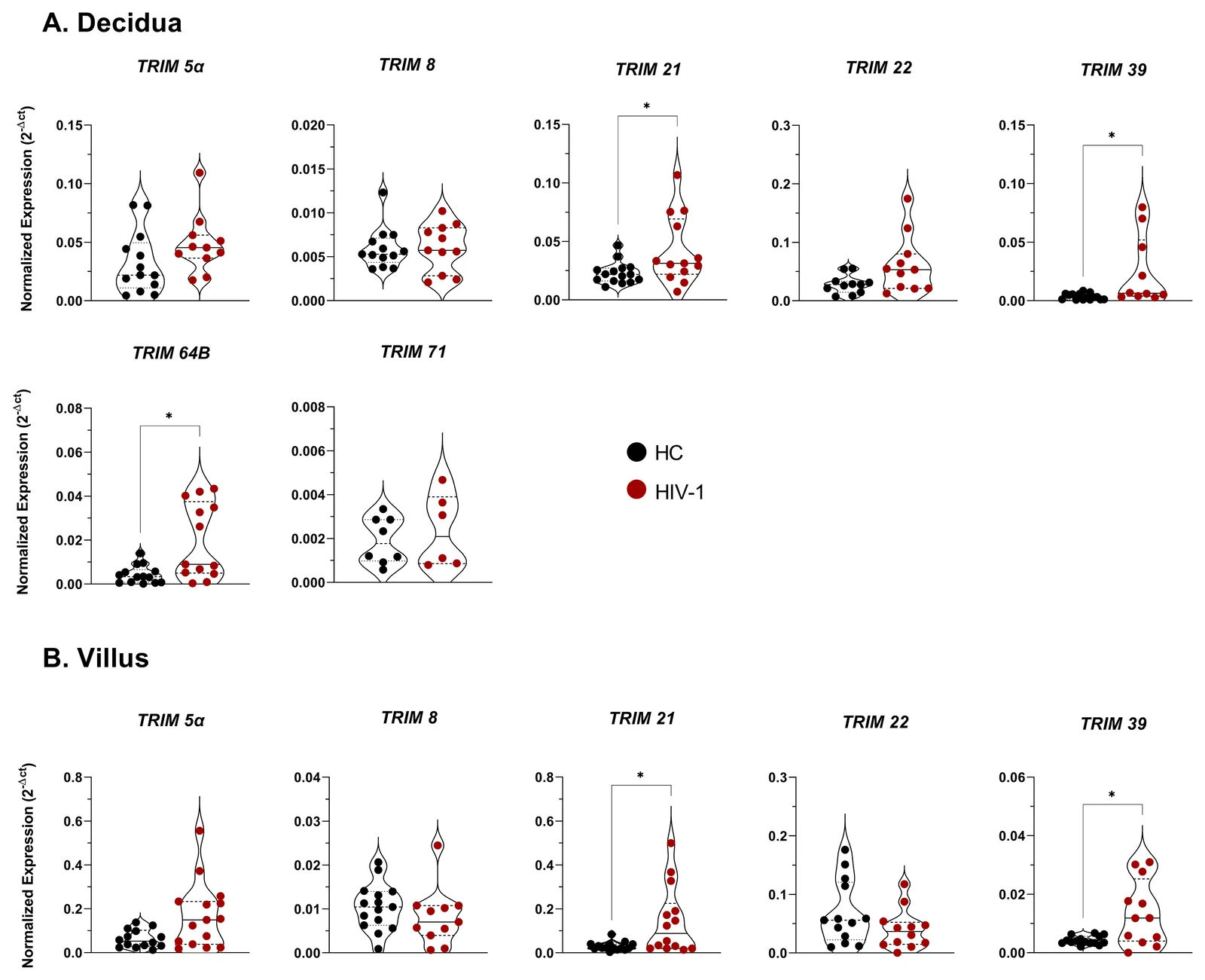
Transcriptional profiling of *TRIM* family genes in the decidual and chorionic villi compartments of HIV-1-infected mothers. Violin plots display the relative mRNA expression levels of *TRIM5α*, *TRIM8*, *TRIM21*, *TRIM22*, *TRIM39*, *TRIM64B*, and *TRIM71* determined via RT-qPCR. A) Placental decidua samples were analyzed from healthy controls (HC, black dots; n = 18) and HIV-1-infected pregnant women under active ART (HIV-1, red dots; n = 15). B) Placental villi samples were analyzed from healthy controls (HC, black dots; n = 18) and HIV-1-infected pregnant women under active ART (HIV-1, red dots; n = 15). Expression levels for *TRIM64B* and *TRIM71* were below the limit of detection within this compartment and are omitted. Individual data points represent independent biological replicates, with horizontal lines indicating the median and interquartile ranges. Relative expression values were normalized using the delta-Ct (2^-ΔCt^) method against reference genes (*GAPDH*, *YWHAZ*). Statistically significant differences between groups were determined using the non-parametric Mann-Whitney U test (*p ≥ 0.05).

In contrast to the decidual profile, the expressions of *TRIM64B* and *TRIM71* were undetectable within the villous compartment, of both control and HIV-1 samples.

### Functional interaction network places TRIM64B, TRIM21 and TRIM39 within innate antiviral pathways despite unaltered SAMHD1 profiles

Due to the upregulation of *TRIM21*, *TRIM39* and TRIM64B, we constructed an interaction network to highlight their associated signaling pathways (**Figure 3**). Gene network was generated using GeneMANIA (25) to identify functionally related genes all genes experimentally assessed in the study. GeneMANIA added 20 functionally associated genes, resulting in an initial network containing 34 genes. Functional enrichment analysis was performed with g:Profiler (26) using all 34 genes in the initial GeneMANIA network. Gene Ontology Biological Process (GO), KEGG and Reactome were included as annotation sources. As expected, the network revealed robust connections with the ubiquitin gene (*UBC*) and the ubiquitin-conjugating enzyme E2 N (*UBE2N*), reflecting that protein modification by ubiquitin is a critical cellular mechanism for targeting abnormal or short-lived proteins for degradation. Several factors as IRF9, ISG15, OAS2, IFIT3, IFITM2 IFITM3 are related with type I interferon pathway. Furthermore, the network enriched pathways critical to the protein ubiquitination, antigen processing: ubiquitination and proteasome degradation innate immune response, including host defense against viruses, cytokine signaling, negative regulation of viral process, interferon alpha/beta signaling, response to virus.

**Figure 3:**
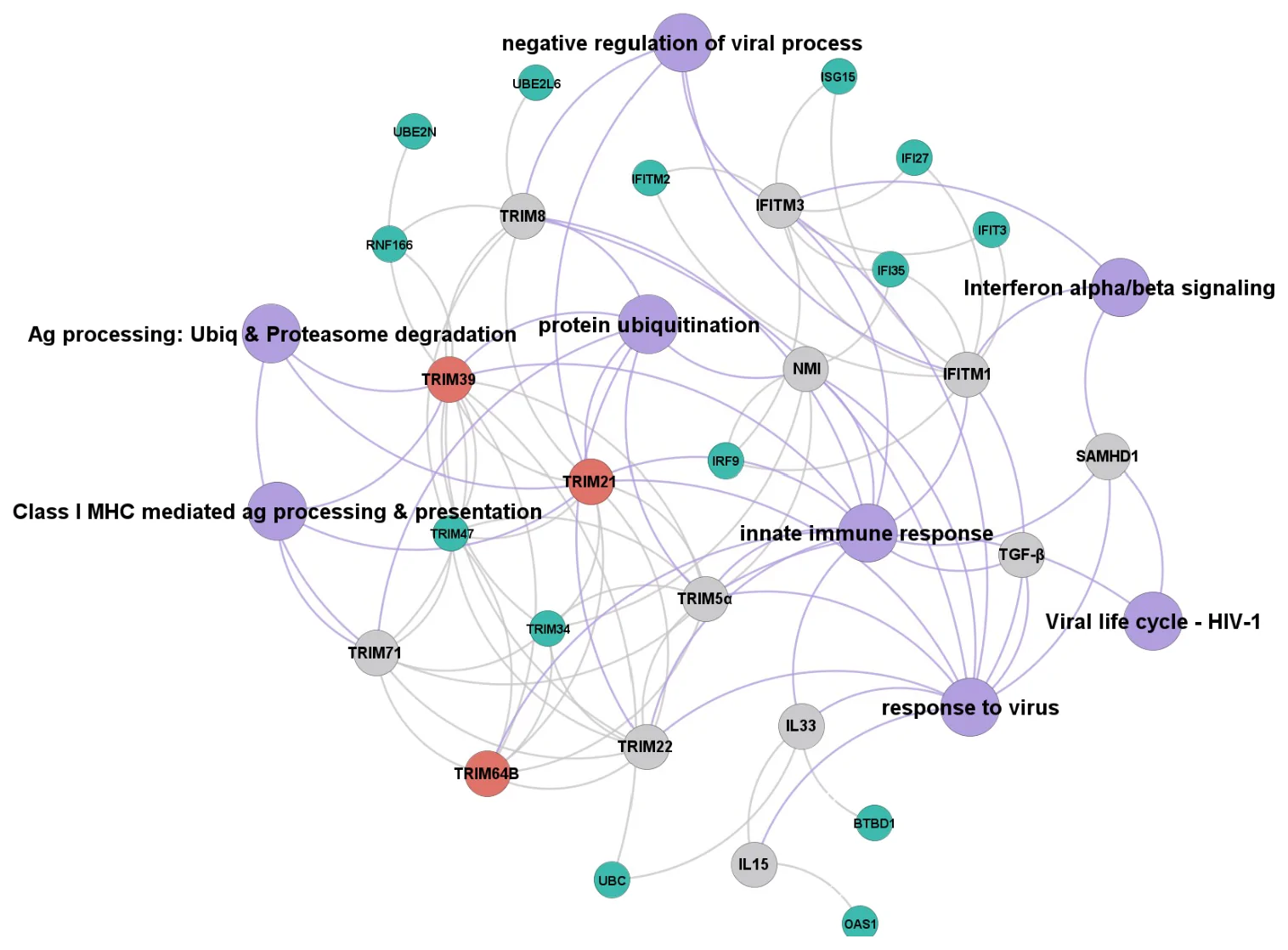
Integrated gene and pathway network of TRIM and innate immune factors. The network integrates functional gene associations identified by GeneMANIA and significantly enriched biological pathways obtained using g:Profiler (adjusted p < 0.05). Red nodes represent experimentally assessed genes that were significantly increased in the HIV-1 group in at least one placental compartment; Purple nodes: enriched pathways; grey nodes: genes experimentally assessed in this study; Node size: degree centrality; Light-grey edges indicate GeneMANIA functional associations, whereas purple edges represent gene–pathway associations identified by g:profiler.

Most TRIM proteins function as E3 ubiquitin-protein ligases with antiviral properties; for instance, TRIM21 engages the Fc region of virus-bound antibodies to restrict viral replication (27, 28). Since TRIM21 has been reported to modulate the expression of the innate antiviral restriction factor SAMHD1 during enterovirus 71 infection (18), we assessed *SAMHD1* expression in the placental villi. However, *SAMHD1* expression remained equilibrated between the HIV-1 and HC groups (**Supplementary Figure 1**). We additionally evaluated other innate immunity genes − including *IFITM1*, *IFITM3*, *IL15*, *IL33*, *TGFB*, and *NMI* (N-Myc and STAT Interactor) − all of which displayed similar expression profiles between the analyzed groups (**Supplementary Figure 1**).

### Enhanced TRIM21 and IgG co-localization unveils an elevated protein complex within the placental villous microenvironment during HIV-1 infection

Driven by the compelling immunological role of TRIM21, we performed immunofluorescence assays to analyze its colocalization with IgG in the villous compartments (**Figure 4**). In the image, we can observe the presence of colocalization (merge) in the zoomed view of the inset (**Figure 4A**). Analysis of the corrected total cell fluorescence revealed that TRIM21 expression was significantly increased in the placental villi of the HIV-1 group compared to HCs (**Figure 4B**). Furthermore, both the intensity of IgG and the amount of colocalized TRIM21-IgG complexes were markedly elevated in the placental villi of HIV-1-infected mothers. In contrast, no significant differences in TRIM21 or IgG expression were observed within the decidual compartment (**Supplementary Figure 2**). Taken together, these findings demonstrate for the first time that the placental villi of HIV-1-infected mothers are enriched with a TRIM21-IgG complex.

**Figure 4:**
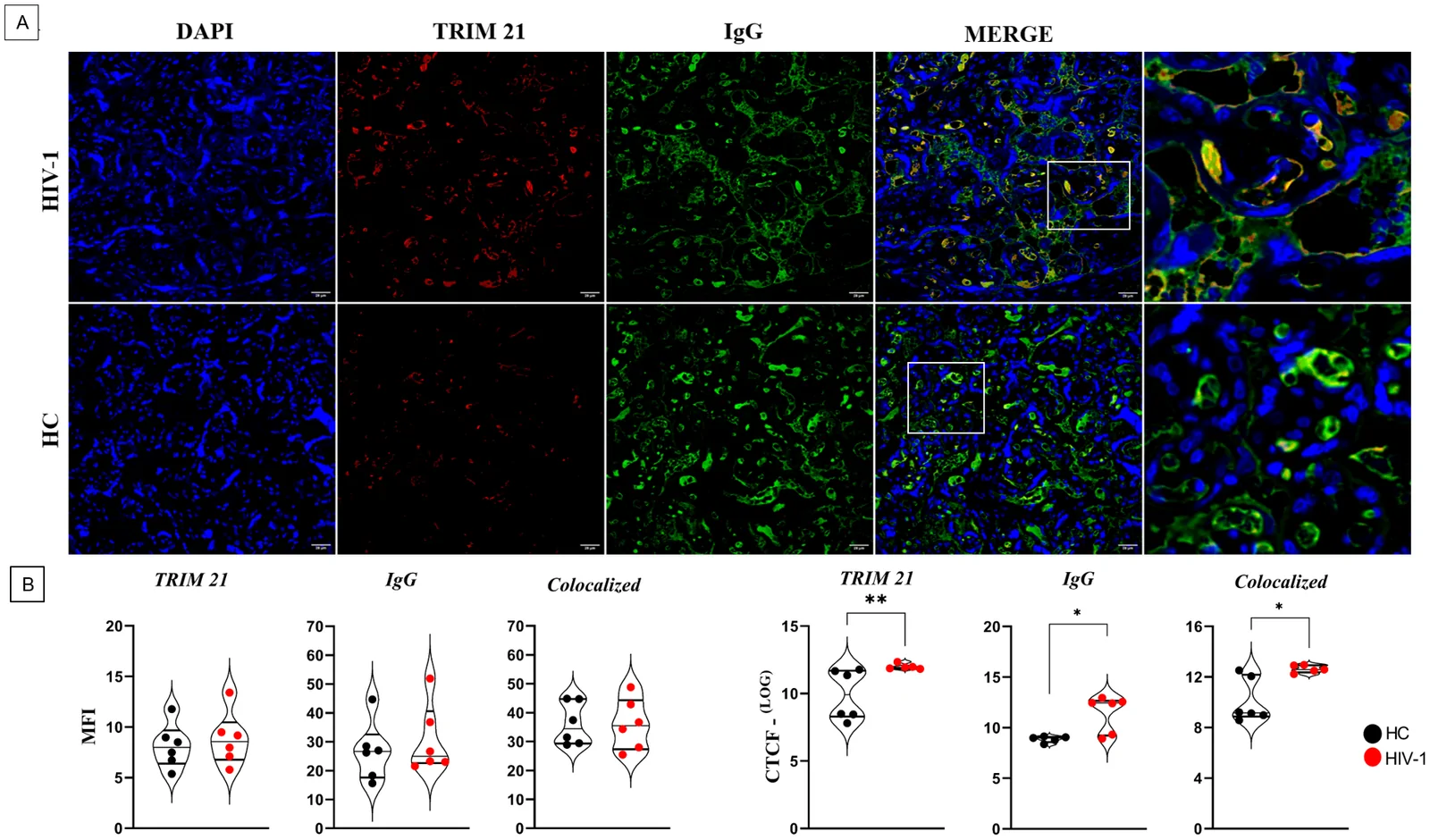
Confocal immunofluorescence images and colocalization of TRIM21 and IgG within the placental villi of HIV-1-infected mothers. **(A)** Representative laser scanning immunofluorescence microscopy images of chorionic villi sections from healthy controls (HC, bottom row) and HIV-1-infected pregnant women (HIV-1, top row). Individual fluorescence channels show cell nuclei counterstained with DAPI (blue), TRIM21 expression (red, Alexa Fluor 633), and maternal IgG distribution (green, Alexa Fluor 555). The merged panel (*Merge*) demonstrates an overlay of the signals, where yellow areas represent the colocalization of TRIM21 and IgG complexes. Scale bars = 28 µm. A zoomed view of the inset is shown on side of the panel. **(B)** Quantitative fluorescence analysis of the villous compartment from healthy controls (HC, n=6) and HIV-1-infected pregnant women (HIV-1, n=6). Scatter plots display individual data points for the Mean Fluorescence Intensity (MFI) and Corrected Total Cell Fluorescence (CTCF) of TRIM21, maternal IgG, and the colocalized TRIM21–IgG complex between the analyzed groups. Horizontal bars represent the mean values. Statistically significant differences were evaluated using the non-parametric Mann-Whitney U test (*p ≥ 0.05, **p ≥ 0.01).

## Discussion

While innate immunity is a critical mechanism protecting the maternal-fetal interface, its precise role in the placenta during chronic, ART-controlled HIV-1 infection remains poorly understood. In this study, histopathological evaluation of placentas from parturient women with undetectable viral loads under ART did not reveal classical inflammatory injuries, such as villitis or deciduitis. However, features of maternal vascular malperfusion (MVM) were clearly evident. Notably, our cohort was devoid of confounding co-infections or major gestational comorbidities, such as pre-eclampsia or gestational diabetes. Both in the pre- and post-ART eras, HIV-1 exposure has been historically linked to placental injury, including MVM and acute or chronic inflammation (29). Our observations align with findings from an uncomplicated pregnant cohort in South Africa, where minimal pathological differences were observed between HIV-infected and uninfected groups, with the notable exception of increased MVM prevalence (30).

Tripartite motif (TRIM) proteins play pivotal roles in regulating the NF-κB pathway through multi-step ubiquitination, thereby modulating innate immunity and exerting robust antiviral effects (14). Here, we demonstrated that both *TRIM21* and *TRIM39* were upregulated across both compartments of the placental tissue from HIV-1-infected mothers. TRIM39, which belongs to the same family as the well-characterized anti-HIV-1 factor TRIM5α, was identified in the placental tissue alongside TRIM5α. While TRIM39 has been implicated in regulating inflammation and cell cycle progression − potentially influencing the delicate immunological balance between maternal and fetal tissues − its direct involvement in HIV-1 infection had not yet been described.

TRIM21 is also recognized as a key regulator of the restriction factor SAMHD1, interacting with it to induce its degradation via the proteasomal pathway. In models of enterovirus 71 (EV71) infection, the virus overcomes SAMHD1-mediated restriction by upregulating host TRIM21, which paradoxically triggers downstream interferon production in neonatal mice (18). In contrast, our study demonstrated that *SAMHD1* transcript levels remained completely intact in the placental tissue of HIV-1-infected mothers. This preservation suggests a protective homeostatic mechanism that prevents the local antiviral integrity of the placenta from being compromised.

The functional network analysis revealed that the interaction partners of TRIM21 and TRIM39 in the placenta are predominantly molecules associated with the ubiquitination machinery, such as ubiquitin-protein ligases and ubiquitin C (*UBC*). Ubiquitination relies on a cascade of three enzyme classes: ubiquitin-activating (E1), ubiquitin-conjugating (E2), and ubiquitin-protein ligase (E3) enzymes. Among these, *UBE2N* encodes an E2 ubiquitin-conjugating enzyme that exerts a dual role during HIV infection; it is hijacked by the virus to facilitate replication, yet it concurrently serves as a host immune sensor to detect viral components (31). For instance, when TRIM5α recognizes the invading HIV-1 capsid, it cooperates with UBE2N to synthesize specific ubiquitin chains. This interaction acts as an intracellular alarm, triggering downstream NF-κB signaling to restrict viral progression. Similarly, the *UBC* gene plays an essential role in maintaining the pool of the ubiquitin-proteasome system, which strictly regulates the HIV-1 life cycle (32).

In addition to the enriched pathways of ubiquitination, interferon-alpha/beta signalling, the innate immune response, the response to viruses and the viral life cycle of HIV-1, as well as the negative regulation of viral processes, were noted. Indeed, interferon-alpha/beta signalling was associated with IFI27 and IFI35, which are both interferon-stimulated genes (ISGs), as well as with IFN-induced proteins containing tetratricopeptide repeats (IFIT proteins), such as IFIT3, and IFN-induced transmembrane proteins (IFITM proteins), such as IFITM1, IFITM2 and IFITM3. These factors have broad-spectrum antiviral functions and are induced by type I interferon (33). Furthermore, ISG15, a small ubiquitin-like protein, acts as an immune defence modifier and can directly inhibit viral replication and modulate host immunity and inflammation (34). NMI encodes a signalling pathway regulator that links cytokine responsiveness to transcriptional control by enhancing STAT1- and STAT5-dependent transcription following IL2 or IFNG stimulation (35). This is a regulatory node in innate antiviral immunity.

Interestingly, we observed an increased, colocalized expression of TRIM21 and IgG within the placental villi, likely mediated through the Fc region of the antibodies. The chorionic villi are continuously bathed in maternal blood, allowing maternal IgG to be transferred to the fetus via neonatal Fc receptor (FcRn)-mediated transcytosis. A limitation of our study is that we could not determine whether the IgG molecules associated with TRIM21 specifically recognize HIV-1 antigens, which would directly promote intracellular ubiquitination and subsequent proteasomal clearance of viral structures. Because our cohort comprised virally suppressed mothers with undetectable viral loads, capturing the physical association between IgG-TRIM21 complexes and active HIV-1 antigens presents a technical challenge.

Maternal HIV infection, even under effective viral suppression, is associated with decreased placental FcRn expression (36), and impaired IgG transfer efficiency across the placenta (37–40). Multiple factors dictate this efficiency, including IgG Fc sub-classifications, glycosylation profiles, and binding affinities to placental FcRs (41). Interestingly, the elevated levels of IgG observed within the placental villi of our HIV-1 group might reflect an accumulation of antibodies resulting from impaired transplacental transfer. Given these dynamics, future investigations should evaluate whether maternal TRIM21 can be transferred to the newborn to participate in neonatal antiviral defense.

In conclusion, our findings demonstrate that the expression of specific TRIM family members is significantly upregulated in placental tissues exposed to controlled HIV-1. We highlight the role of TRIM21, which potentially engages the Fc region of antigen-bound maternal IgG within the villi to favor intracellular ubiquitination and signaling. By directly interacting with target complexes or modulating inflammatory cascades, TRIM21 emerges as a key player at the maternal-fetal interface, offering promising avenues for future therapeutic exploitation.

## Materials and methods

### Study population and sample collection

Participants in this study comprised pregnant women living with HIV-1, with gestational ages ranging from 35 to 40 weeks (n = 15), recruited from the obstetrics outpatient clinics of the Hospital das Clínicas da Faculdade de Medicina da Universidade de São Paulo (HC/FMUSP) and the Centro de Atendimento Integral à Saúde da Mulher (CAISM) at the State University of Campinas (UNICAMP). All HIV-1-infected mothers underwent elective cesarean sections. Mothers who were not adhering to ART and/or whose adherence varied during pregnancy were given intrapartum dosing protocol therapy (intravenous zidovudine – AZT) 3–5 hours before delivery. The ART used during pregnancy and the clinical and demographic characteristics are described in **Table 1**. The control group consisted of clinically healthy parturient women (n = 18), from elective caesarean section, with gestational ages ranging from 38 to 41 weeks, who demonstrated negative serology for HIV-1, syphilis, hepatitis B and C, toxoplasmosis, and other infectious diseases (**Table 2**). Control samples were obtained from the Maternity Hospital of the University Hospital (HU/USP). This study was approved by the Institutional Ethics Committees of the University of São Paulo and the University of Campinas (CAAE: 96994618.2.0000.0065). Written informed consent was obtained from all participants or their legal guardians prior to enrollment.

### Histopathology and morphological analysis

Placental tissue biopsies were fixed, embedded in paraffin, and cut into 4-µm thick sections before being mounted on silanized slides (Sigma Chemical Co., St. Louis, MO, USA). Sections were deparaffinized in xylene baths and subsequently rehydrated through a graded series of ethanol concentrations. Slides were stained with hematoxylin and eosin (H&E) for conventional morphological analysis. Histopathological parameters were evaluated under a light microscope by an experienced pathologist in a blinded manner.

### RNA extraction and quantitative real-time PCR

Total RNA extraction was performed on cryopreserved placental decidua and chorionic villi specimens. Tissues were mechanically disrupted using a TissueRuptor (Qiagen, Hilden, Germany), and total RNA was isolated using the RNeasy Plus Mini Kit (Qiagen) according to the manufacturer’s protocol. RNA concentration and purity were quantified using a NanoDrop ND-1000 spectrophotometer (Thermo Fisher Scientific, Waltham, MA, USA). Reverse transcription of total RNA was performed using a commercial Reverse Transcriptase Kit (Bio-Rad, Berkeley, CA, USA) following the manufacturer’s instructions. Quantitative real-time PCR (RT-qPCR) amplification reactions were conducted on an Applied Biosystems 7500 Real-Time PCR System (Thermo Fisher Scientific). Each reaction utilized 5 ng/µL of cDNA, 1 µM of gene-specific primers, and the Applied Biosystems SYBR Green PCR Master Mix (Thermo Fisher Scientific).

The gene expression levels were normalised using the internal reference genes GAPDH and YWHAZ. A media performance was then carried out. Primer sequences for all target genes are detailed in **Supplementary Table 1**. Data analysis was performed using the 7500 Software v2.0.6 (Applied Biosystems) based on the relative quantification de 2-ΔΔCt method (42).

### Functional interaction and pathway network analysis

Gene network was generated using GeneMANIA (25) to identify functionally related genes all genes experimentally assessed in the study. GeneMANIA added 20 functionally associated genes, resulting in an initial network containing 34 genes. Functional enrichment analysis was performed with g:Profiler (26) using all 34 genes in the initial GeneMANIA network. Gene Ontology Biological Process (GO), KEGG and Reactome were included as annotation sources. Statistical significance was determined using the Benjamini–Hochberg false discovery rate (FDR) correction, and only pathways with an adjusted p-value < 0.05 were considered significant and incorporated into the network.

### Immunofluorescence and image analysis

Placental tissue sections were deparaffinized in xylene, rehydrated through a graded ethanol series, and incubated with 10% hydrogen peroxide to block endogenous peroxidase activity. For dual immunofluorescence labeling, sections were incubated overnight at 4 °C with a rabbit anti-human IgG Fc antibody (Thermo Fisher Scientific) and a goat anti-TRIM21/SS-A primary antibody (Abcam, Cambridge, UK). After followed by two steps of incubation one with donkey anti-goat IgG antibody conjugated to Alexa Fluor 633 (Invitrogen, Carlsbad, CA, USA) and other with goat anti-rabbit conjugated to Alexa 555 (Abcam) for 1 hour at room temperature. Nuclei were counterstained with DAPI for 2 minutes. Slides were mounted using a glycerol-based mounting medium.

High-resolution automated image acquisition was performed using a TissueGnostics scanning confocal microscope equipped with a 20× objective lens at the Center for Facilitation of Research and Fluorescence Assessment (CEFAP-USP). Primary image processing was conducted using StrataQuest 8.0.1 software (TissueGnostics, Vienna, Austria). Quantitative colocalization and fluorescence intensity analyses were performed using Fiji/ImageJ software. Raw TIFF images containing triple-color signals were split into individual channels and assigned pseudo-colors (DAPI for nuclei, anti-IgG in green, and TRIM21 in magenta). Following background subtraction, signal thresholds were automatically determined using the Otsu method to define positive areas for each marker. Colocalization regions were isolated by generating binary masks for each channel and applying the “Image Calculator - AND” function to map overlapping IgG and TRIM21 signals. Integrated density and area measurements within the Regions of Interest (ROIs) – manually outlined based on tissue morphology (decidua vs. villi) – were acquired using the ROI Manager. The Corrected Total Cell Fluorescence (CTCF) was calculated using the standard formula: CTCF = Integrated Density − (Area of ROI × Mean fluorescence of background readings) (43). The final composite images were combined and exported in PNG format for the assembly of the figure panel, using the image processing software FigureJ (Fiji) (44). For the quantitative immunofluorescence analysis, one section from each patient was analyzed. This section comprised three color channels (red, green, and merged), each of which was analyzed separately. This procedure was applied to both the villous and decidual regions.

### Statistical analyses

Intergroup statistical comparisons were performed using the non-parametric Mann-Whitney U test. Statistical significance was established at *p*-value ≤ 0.05. All statistical computations and graphing were performed using GraphPad Prism software.

## Data Availability

The datasets generated and/or analyzed during the current study are not publicly available due to ethical and privacy restrictions related to the use of human placental samples and sensitive clinical data, but are available from the corresponding author on reasonable request.
