# Supplementary material for: Enrichment of the TRIM21-IgG complex in placental villi during HIV-1 infection
Source: Front Immunol. 2026 Sep 18;17:1922263. doi: 10.3389/fimmu.2026.1922263 (PMC13631741; doi:10.3389/fimmu.2026.1922263)
Supplement: Supplementary file 1 [file DataSheet1.docx]

Supplementary Material

## Supplementary Figures

**Supplementary Figure 1. Transcriptional expression of innate immune factors in the decidual and chorionic villi compartments of HIV-1-infected mothers.** Scatter dot plots display the relative mRNA expression levels of *IFITM1*, *IFITM3*, *IL15*, *IL33*, *TGFB*, *NMI*, and *SAMHD1* evaluated via RT-qPCR within the **(A)** decidua (top row) and **(B)** chorionic villi (bottom row). Placental tissue samples were analyzed from healthy controls (HC, black dots; n = 18) and HIV-1-infected pregnant women under active ART (HIV-1, red dots; n = 15). Individual data points represent independent biological replicates, with horizontal lines indicating the median and interquartile ranges. No statistically significant differences were observed between the analyzed groups for any of the targets evaluated. Relative expression values were normalized using the delta-Ct (2^-∆Ct^) method against the reference genes *GAPDH* and *YWHAZ*. Intergroup comparisons were evaluated using the non-parametric Mann–Whitney U test (p > 0.05).

**Supplementary Figure 2. Confocal immunofluorescence analysis and quantitative profiling of TRIM21 and IgG within the decidual compartment.** Representative laser scanning immunofluorescence microscopy images of placental decidua sections from healthy controls (HC, bottom row) and HIV-1-infected pregnant women (HIV-1, top row) (n = 6 per group). Individual fluorescence channels display cell nuclei counterstained with DAPI (blue), TRIM21 expression (red, Alexa Fluor 633), and maternal IgG distribution (green, DyLight 550). The pink dashed lines delineate the specific morphologically defined maternal decidual zones manually selected for region-specific quantification. The merged panel (*Merge*) demonstrates overlapping signals, where yellow areas represent the localized intracellular co-occurrence of TRIM21 and IgG. Scale bars = 28 µm. Quantitative fluorescence analysis restricted to the decidual ROIs. Scatter plots show individual data points for the Mean Fluorescence Intensity (MFI) and Corrected Total Cell Fluorescence (CTCF) of TRIM21, maternal IgG, and the colocalized TRIM21-IgG complex between the studied groups. Horizontal lines indicate the mean values. No statistically significant differences were detected between the HC and HIV-1 groups within this anatomical compartment using the non-parametric Mann–Whitney U test (p > 0.05).

## Supplementary Tables

**Supplementary Table 1**. Oligonucleotides used in real-time PCR experiments

| **Target** | **Sequence Forward 5’– 3’** | **Sequence Reverse 5’– 3’** |
| --- | --- | --- |
| ***TRIM 5α*** | CTGGCATCCTGGGCTCTCAAAGT | CATACCCCCAGGATCCAAGCAGTT |
| ***TRIM 8*** | ATCCTGATGGACAGGACCCA | CTCCTTCTTGGCCACTTCGT |
| ***TRIM 21*** | TCAGCAGCACGCTTGACAAT | GGCCACACTCGATGCTCAC |
| ***TRIM 22*** | GGTTGAGGGGATCGTCAGTA | TTGGAAACAGATTTTGGCTTC |
| ***TRIM 39*** | AAGGTGAAGACCATGGAGGTGACT | TAGGTTAGGATGAGCTGTCTCAGGGT |
| ***TRIM 64B*** | GAGAGCTGTGGGAGACATGC | CTGTGCCAAATCAGTCCTTGC |
| ***TRIM 71*** | AACAGGTGGAGATGAAGGCG | GGCGGATCTTTTCTACCTTCCA |
| ***IL 15*** | AACAGAAGCCAACTGGGTGAATG | CTCCAAGAGAAAGCACTTCATTGC |
| ***IL 33*** | CACCCCTCAAATGAATCAGG | GGAGCTCCACGAGTGTTCC |
| ***IFITM 1*** | CGCCAAGTGCCTGAACATCT | TACCAGTAACAGGATGAATCCAATG |
| ***IFITM 3*** | CAAGGAGGAGCACGAGG | TTGAACAGGGACCAGACG |
| ***TGF-β*** | GGAAATTGAGGGCTTTCGCC | AGTGAACCCGTTGATGTCCA |
| ***NMI*** | AAGGAGCATTCGCCAGATGAA | GTAGCCTCTTGTAACTCCGTTTC |
| ***SAMHD-1*** | GGATTACTAAAAACCAGGTTTCACAACT | GCTCTGCAAATTTCTCTGGCAG |
| ***GAPDH*** | GAAGGTGAAGGTCGGAGT | GAAGATGGTGATGGGATTTC |
| ***YWHAZ*** | GCCACAATGTTCTTGGCCCATCAT | TGGTTGGTGACAAGACAGAAGGCT |
